# BAP1 regulates different mechanisms of cell death

**DOI:** 10.1038/s41419-018-1206-5

**Published:** 2018-11-19

**Authors:** El Bachir Affar, Michele Carbone

**Affiliations:** 1 Maisonneuve-Rosemont Hospital Research Center, Department of Medicine, University of Montréal, Montréal, Quebec, H1T 2M4 Canada; 2 Hawaii Cancer Center, Honolulu, Hawai’i 96813 USA

The ubiquitin carboxyl terminal BAP1 is a member of deubiquitinating enzymes superfamily, which are responsible for coordinating ubiquitin-signaling processes through the removal of ubiquitin from protein substrates. Studies of families with high incidence of mesothelioma^1,2^, led to the discovery that all affected family members carried heterozygous BAP1 mutations (BAP1^+/−^)^3^, a condition that was named “the BAP1 cancer syndrome”^4,5^. Since the initial discovery in 2011, over 100 families have been identified worldwide affected by the BAP1 cancer syndrome. Thus far, all carriers of heterozygous germline BAP1 mutations have developed one or more malignancies during their lifetime^5,6^.

The powerful tumor suppressor activity of BAP1 and its ability to regulate gene × environment interactions in carcinogenesis has been linked to its dual role in the nucleus and in the cytoplasm. In the nucleus, BAP1 modulates the transcriptional regulation of several gene programs and promotes DNA repair by facilitating homologous recombination^7–9^.

In the cytoplasm, BAP1 regulates Ca^2+^-signaling mediated cell death. Bononi et al.^10^ reported that BAP1 deubiquitylates and stabilizes the IP3R3 channel-receptor. This channel regulates the release of Ca^2+^ from the endoplasmic reticulum (ER) into the cytoplasm in areas known as mitochondrial associated membranes where the ER and mitochondria are in close proximity. Ca^2+^ released from the ER moves into the mitochondria through the voltage-dependent anion channels that are located on the outer mitochondrial membrane, and then through the mitochondrial Ca^2+^  uniporter, which are the channels that allow Ca^2+^ to cross the inner mitochondrial membrane. Ca^2+^ is required by several enzymes that regulate mitochondrial respiration (Krebs cycle); but high levels of mitochondrial Ca^2+^ promote cytochrome *c* release from the mitochondria into the cytoplasm, a process that leads to apoptosis^10^. In summary, reduced BAP1 levels cause cells to accumulate DNA damage, a process that normally triggers apoptosis. However, the reduced amount of BAP1 in the cytoplasm of BAP1^+/−^ cells impairs apoptosis and allows survival of cells that have acquired oncogenic mutations. Moreover, reduced BAP1 levels in “normal” cells, induce a Warburg effect, i.e., a shift from oxidative phosphorylation (Kreb’s cycle) to aerobic glycolysis, a process that might prime these “normal” cells for malignant transformation and tumor growth^11^. These findings provided an explanation for the potent tumor suppressor activity of BAP1 and for the prevalence of cancers associated with exposure to environmental carcinogens.

Recently, Zhang et al.^12^ reported in *Nature Cell Biology*, a novel mechanism by which BAP1 regulates cell death. The authors demonstrated that BAP1 regulates ferroptosis by repressing the expression of SLC7A11 gene leading to low levels of reduced glutathione and diminished antioxidant capacity of the cells.

## BAP1 tumor suppression might involve its ability to inhibit ferroptosis

**Fig. 1:**
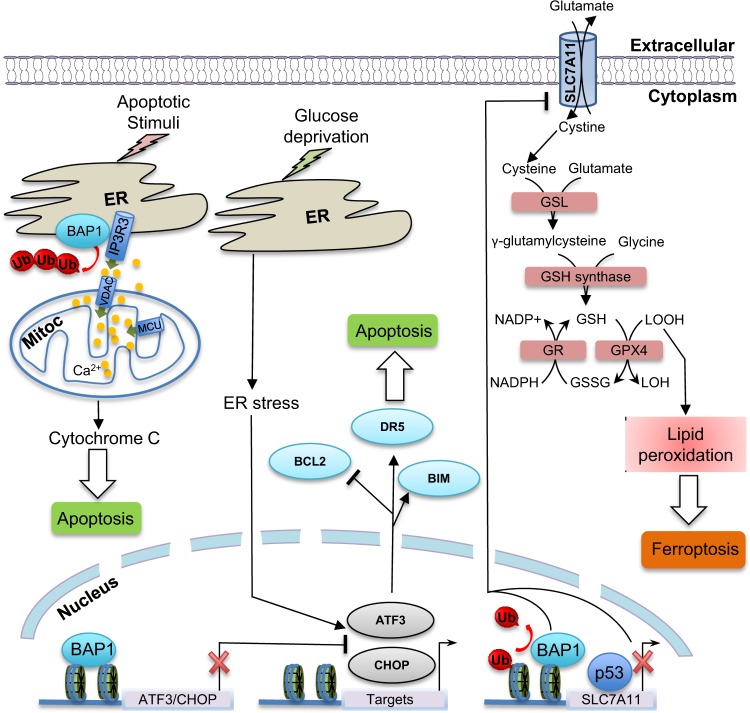
Roles of the deubiquitylase and tumor suppressor BAP1 in cell death. The tumor suppressor BAP1 promotes ferroptosis by repressing the expression of SLC7A11, a cystine/glutamate antiporter responsible for the cellular entry of cysteine, a metabolite used for the novo synthesis of reduced glutathione. Inhibition of SLC7A11 expression leads to low levels of reduced glutathione, diminished antioxidant capacity of the cells, thus resulting in Lipid-ROS accumulation and ferroptosis. BAP1 deubiquitylates and stabilizes the IP3R3 channel-receptor, thus promoting the release of Ca^2+^ from the endoplasmic reticulum (ER) into the cytoplasm and mitochondria (Mitoc). High levels of mitochondrial Ca^2+^, promote cytochrome c release from the mitochondria into the cytoplasm, a process that leads to apoptosis. Therefore, low levels of BAP1 inhibit both apoptosis and ferroptosis, facilitating the survival of DNA damage cells. BAP1 can also inhibit apoptosis by repressing the expression of key ER stress transcription factors with pro-apoptotic functions. The ability of BAP1 to modulate apoptosis and ferroptosis contributes to its tumor suppressor function in vivo. GSL: glutamate-cysteine ligase; GSH: Reduced glutathione; GSSG: oxidized glutathione; GR: Glutathione Reductase; GPX4: Glutathione Peroxidase 4; VDAC: Voltage-dependent anion channel; MCU: mitochondrial Ca^2+^ uniporter; Ub: Ubiquitin

Zhang et al.^12^ conducted an unbiased genome-wide analysis of monoubiquitinated H2A on lysine 119 (hereafter, H2Aub) following BAP1 expression in BAP1-deficient renal cancer cells. As the expression of BAP1, but not its catalytic dead form, is known to reduce the global levels of H2Aub, this strategy has been used to capture genomic locations that exhibit higher levels of H2Aub using chromatin immunoprecipitation in combination with next-generation genome sequencing (ChIP-Seq)^12^. Few thousands genomic locations have been inferred as potentially regulated by BAP1 owing to changes of H2Aub  levels. These genes were integrated with differentially expressed genes identified, in parallel, by RNA sequencing (RNA-Seq) following BAP1 expression. This resulted in a reduced list revealing that BAP1-downregulated genes were enriched in cell metabolism-associated genes. Bioinformatics revealed SLC7A11 as a BAP1-downregulated gene whose expression inversely correlated with BAP1, suggesting a potential link between BAP1, cystine uptake, antioxidant capacity, and ferroptosis^12^. These observations prompted the team to investigate the functional relationship between BAP1 and SLC7A11. SLC7A11 appears to be a direct target of BAP1, which reduces the levels of H2Aub on SLC7A11 promoter, but yet represses its transcriptional activity. BAP1 suppresses SLC7A11-mediated cystine uptake resulting in decreased pools of reduced glutathione, increased lipid peroxidation thereby promoting ferroptosis^12^. BAP1 effects on ferroptosis seem independent of p53 expression, suggesting that these two tumor suppressors use different routes to modulate this form of cell death (Figure 1). Ferroptosis appears to be relevant to BAP1 tumor suppressor function and BAP1 mutations that target its catalytic site are defective in regulating SLC7A11 expression and are unable to promote ferroptosis.

## BAP1 might exemplify a new network of ferroptosis regulation

BAP1 is subjected to multiple signaling cascades that impart several post-translational modifications on this DUB including phosphorylation and ubiquitination, both of which coordinate BAP1 function^9,13^. Hence, it will be interesting to determine whether stimuli and stress conditions triggering signaling pathways upstream BAP1 can result in the modulation of SLC7A11 expression and the threshold of ferroptosis. Moreover, H2A ubiquitination is regulated by the Polycomb group complex PRC1, and components of this complex, BMI1 and RING1B, are often overexpressed in cancer, raising the possibility that overactivation of PRC1 might lead to increased SLC7A11 expression, increased antioxidant capacity, and protection against ferroptosis. Notably, BMI1 have been involved in oxidative stress responses and appears to regulate cell death, at least indirectly, through expression of SLC7A11. It will be important to study the possible interplay between BMI and BAP1 in coordinating ferroptosis. Do BAP1-associated components impact BAP1 function in mediating ferroptosis? ASXL1 and ASXL2, frequently mutated in cancer, facilitate BAP1 DUB activity^14,15^. Thus, it is predicted that depletion of these factors would mimic BAP1 inactivation, resulting in increased SLC7A11 expression and increased threshold for triggering ferroptosis. However, depletion of ASXL1, but not ASXL2, reduced SLC7A11 expression^12^. Two other major components of the BAP1 complexes, HCF-1 and OGT, are also of interest, and while depletion of HCF-1 does not seem to impact SLC7A11 expression, depletion of OGT does^12^. Taking into account that OGT is essentially brought to the BAP1 complex via HCF-1^15^, this might suggest that another pool of OGT might, independently of BAP1, regulate SLC7A11 expression. Depletion of BAP1-associated lysine demethylase, KDM1, results in increased SLC7A11 protein levels, whereas depletion of the two transcription factors FOXK1 and FOXK2 reduces SLC7A11 levels, suggesting that BAP1-associated proteins play either analogous or opposite roles in regulating SLC7A11 expression. These results are intriguing and suggest that, although BAP1 is a multimeric complex with several activities involved in its recruitment and control of its activity, some associated partners might impose another layer of regulation on BAP1-mediated ferroptosis. Although this remains to be investigated, these results, suggest that the majority of BAP1 partners regulate SLC7A11 expression and possibility impact ferroptosis.

Because ferroptosis and apoptosis are two distinct programs in BAP1-mediated cell death, it will be worth to investigate whether these processes act in concert or independently during cancer development. It is possible that, depending on the cellular context and the stages of malignant transformation, BAP1 might mandate one or both of these processes. BAP1 and p53 are possibly the two most potent tumor suppressor genes, as they are the only genes that, when heterozygously mutated in the germline will cause one or more cancers in nearly all affected carriers. Both, BAP1 and p53 regulate transcription and DNA damage/repair responses, modulate cell death programs and coordinate cell metabolism. Is there a cross-talk between these factors during development and human cellular transformation? These remain outstanding questions to address.
